# Effectiveness of prime vendor system on availability of medicines and medical supplies in the selected public health facilities in Arusha district council

**DOI:** 10.1186/s12913-024-10581-4

**Published:** 2024-02-01

**Authors:** Liberatus Elias, Lawrencia Mushi

**Affiliations:** 1 Now For Compassion (NFC) Tanzania, Arusha, United Republic of Tanzania; 2 Department of Health Systems Management, School of Public Administration and Management, Mzumbe University, Morogoro, United Republic of Tanzania

**Keywords:** Effectiveness, Prime vendor system, Prime vendor, Medicines, Medical supplies, Public health facilities

## Abstract

**Introduction:**

The shortage of medicines and medical supplies remains to be a major issue that is facing public health facilities in Tanzania. This situation has been influenced by lack of consistency in the supply chain, increase in healthcare demand, poor regulatory system, insufficient funds, and lack of conducive infrastructure. Formerly, the Government initiatives such as engagement with the Prime Vendor System (PVS) demonstrated great assistance in getting rid of this challenge. Despite the operation of PVS, a recent shortage of medicines and medical supplies has been noticed.

**Objectives:**

This study aimed to assess the effectiveness of PVS on the availability of medicine and medical supplies in the selected public health facilities in Arusha District Council.

**Methods:**

The study used a case study design with mixed research approach. The study involved 77 respondents which included 25 health facility in-charges, 50 patients, 1 District Pharmacist and 1 Prime Vendor. Questionnaires, interviews, and observation methods were used to collect data. Data collected covered a period of 2021–2022. Thematic analysis was used to analyze the qualitative data whereas descriptive analysis was used to analyze the quantitative data with the help of Excel and the Statistical Package for Social Sciences (SPSS) version 28.0.

**Results:**

The analysis indicates that PVS is not completely effective in supplying medicines and medical supplies due to its low capacity to conform to the orders placed by the public health facilities, a lack of supply competition, and a failure to adhere to contractual terms. Furthermore, at the time of data collection, the average availability of medicines and medical supplies at the selected public health facilities was 74.8%, while 80% of the selected public health facilities reported having a scarcity of medicines and medical supplies, and 92% of the interviewed patients reported having no full access to medicines.

**Conclusion:**

Despite the shortcomings associated with the operation of the PVS, the system still seems to be very important for enhancing the availability of medicines and medical supplies once its effectiveness is strengthened. This study recommends a routine monitoring of PVS operations and timely interventions to reinforce an adherence to the contracted terms and improve PVS effectiveness.

## Introduction

Medicines and medical supplies are important commodities that should be adequately available in all health facilities [1]. These commodities have a significant role in saving human lives, improving the health status of the community, and strengthening the health system [2]. The availability of medicines and medical supplies at health facilities does not only make health care providers confident, but also it allows patients to be accessed with the complete medical treatments [3]. However, shortage of medicines and medical supplies remains to be a major issue that is facing public health facilities in Tanzania [4–6]. This situation has been influenced by lack of consistency in the supply chain, increase in healthcare demand, poor regulatory system, insufficient funds, and lack of conducive infrastructure [7–10]. Formerly, the Government initiatives such as engagement with the Prime Vendor System (PVS) demonstrated effective assistance in getting rid of the challenge of shortage of medicines and medical supplies [11].

The concept of “Prime Vendor System” is not a new term in the field of health care supply chain. Several countries around the world have been implementing PVS to enhance their procurement processes [12]. It is the contracting procuring system that is involved in purchasing medicines and medical supplies from multiple suppliers and collecting them together for delivery to the health facilities based on the placed orders [13]. Prime vendors engage in buying medicines and medical supplies from various sources or suppliers, putting them on a centralized platform, and finally making deliveries to a range of health facilities [14]. The system allows public health facilities to obtain medicine and medical supplies from a single source that is a prime vendor.

In 2014, the health system of Tanzania started to partner with the PVS, called the JAZIA Prime Vendor System (Jazia PVS) [15]. Jazia PVS began in three regions: Dodoma, Morogoro, and Shinyanga, before being expanded to all regions of Tanzania in 2018 [11]. Engagement with the PVS intended to complement the supply gaps left by the Medical Stores Department (MSD) which is an autonomous department within the Ministry of Health [15]. For each region, there is only one prime vendor who is selected regarding on the price of medicines and medical supplies; financial stability; manpower; transport means; quality assurance; procurement processes; and warehouses [16]. After selection, the prime vendor is contracted for two years to operate in a specific region.

Public health facilities are obliged to buy required medicines and medical supplies from the contracted prime vendors after receiving out-of-stock (O/S) reports from MSD. The O/S reports are provided on a quarterly basis to notify the health facilities of the missing items that were supposed to be supplied. Once the health facilities have received the O/S reports, the health facility in-charges are supposed to place the orders to the prime vendors for the missing items as soon as possible [11].

Implementation of PVS influenced the increase in availability of medicines and medical supplies from 69% in 2014 to 94% in 2018 [15]. This was a great achievement which contributed to strengthening the health system in Tanzania [11]. The operation of PVS provided a room for price comparison to determine the most cost-effective source, shortened the delivery time, minimized losses and enhanced good efficiencies of the health system [17].

However, recent studies conducted in Tanzania reported the existence of shortage of medicines and medical supplies in the public health facilities especially those located in rural areas [18, 19]. Therefore, despite the operation PVS, the problem of shortage of medicines and medical supplies to the public health facilities has not yet been addressed. Therefore, as part of addressing of the existing knowledge gap realized from previous studies, the current study assessed the effectiveness of the PVS on the availability of medicines and medical supplies in public health facilities.

## Methods

### Study area

The study was conducted in Arusha District Council. This area was chosen conveniently as the area of the study due to the fact that all district councils use the same system of acquiring medicines and medical supplies from prime vendors. Arusha District Council is one of the seven districts of the Arusha Region of Tanzania.

### Study design, population, and sampling

The study used a case-study design. The use of a case study design was due to the fact that there was a phenomenon to be observed, which is the effectiveness of PVS. The study involved 77 respondents in which 25 were health facility in-charges, 50 were patients, 1 was the District Pharmacist and 1 was the Prime Vendor.

Purposive sampling was used to select study respondents. A sample size of 77 respondents was obtained as follows: For the health-facility-in charges, Yamane 1973 formula was used to obtain a sample of 25 respondents from a population of 34 health facility in-changes. For patients: A sample of 50 patients was obtained from 25 selected public health facilities whereby 2 patients from outpatient department were selected. The study opted to involve only outpatients because, at the time of data collection, 76% of the selected public health facilities were running outpatient clinics. The time limit influenced the decision to interview only 2 patients from each health facility.

Public health facilities were included if they outsource medicines and medical supplies from the PVS. All health facility in- charges from the selected public health facilities were included because they are responsible for all issues of procurement of medicines and medical supplies. The study also included the contracted the Prime Vendor from Arusha region who is responsible for complementing medicines and medical supplies to the public health facilities and the District Pharmacist who oversees stock status of medicines and medical supplies in the district. Two patients who regularly visited a particular health facility were included in the study.

The study excluded all health workers that were not familiar with the operation of the PVS and all patients who had no regular visit and/and those who have visited a health facility for the first time.

### Data collection and measurements

This study used primary source of data which directly obtained from the respondents by using questionnaires and interviews.

Questionnaires with both structured and unstructured questions were administered to health facility in- charges, district pharmacist and prime vendor. The approach was suitable to these respondents because all of them were able to read and write, and had limited time for the interview. Questionnaires were intended to gather information concerning the current status of availability of medicines and medical supplies, capacity of prime vendor in fulfilling medicine and medical supply orders, existence of any competitors against the contracted prime vendor and adherence to the contracted terms and conditions. Questionnaires were printed on the A4 white paper and physically delivered to the office of each respondent. A time-frame of one to two weeks was used to distribute and collected the filled questionnaire forms.

Interviews were conducted for all selected patients, and they were asked both structured and unstructured questions. Patients were included in this study because they are the final consumers of medicines; hence, they were also right respondents to give out information on the accessibility of medicines in the public health facilities Questions were read to patients by the interviewer because most of them were not able to read and write. Face- to- face interviews were conducted by using Swahili language and sometimes Maasai language was used in order to enable respondents to understand interview questions. The interview for each respondent lasted for 2 to 5 min. Interview notes were taken in the notebook and also recorded using a voice recorder.

### Data analysis

The study used both qualitative and quantitative data. Thematic analysis was used to analyze qualitative data. The method involved six steps: familiarization with the data, coding, generating themes, reviewing themes, defining, and naming themes, and writing up. Familiarization involved passing through the collected data to learn the collected information. Coding data involved highlighting the section and coming up with shorthand labels or codes to describe the contents represented. The generation of themes focused at all the codes that were created, and then the codes were related to one another to generate a theme of codes. For the effective reviewing of themes at this point, all created themes were carefully evaluated for their accuracy in representing the desired information. Defining and naming themes: this was another step whereby all themes were defined by indicating what exactly they were meant for; naming themes involved assigning understandable names to each theme. The final step was writing up a report.

Descriptive analysis was used to determine the frequencies average and percentages. The tools used were Excel and Statistical Package for Social Sciences (SPSS) version 28.0 (IBM SPSS Statistics 28.0), which enabled the generation of tables to examine the findings.

The findings from the data analysis were presented using tables and descriptions.

## Results

### Demographic characteristics of all participants

Demographic characteristics considered in this study were sex, education, and location of respondents. For the sex category; 67.5% were females and 32.5% of respondents were males. In terms of respondents’ education levels, 5.2% of them had attained university education, 29.9% of the respondents had attained college education, 10.4% of them had attained secondary education, 28.6% of them had attained primary education and 25.9% of the respondents had not attended any education level. Majority of respondents 58.4% were from rural areas while 41.6% were from urban areas. All respondents were above 18 years old.

### The status of availability of medicines and medical supplies

The study sought to understand the status of availability of medicines and medical supplies at the selected public health facilities. By enhancing this, the assessments were focused on determining the current average of availability of medicines and medical supplies; increased availability of medicines and medical supplies; stock availability; challenges of shortage of medicines and medical supplies and patients access to essential medicines.

Assessment on the current average of availability of medicines and medical supplies to public health facilities, the items were reported in general without considering specific types of medicines and medical supplies. The findings obtained from 25 health facility in-charges were as follows: 8 respondents reported the current average availability of medicines and medical supply to be 90%, 15 respondents reported to be 70% and 2 respondents reported the current average availability of medicines and medical supply to be 50%. However, the overall current availability of medicines and medical supplies at the selected public health facilities was determined to be 74.8%. Table 1 presents the average availability of medicines and medical supplies in each selected public health facility, as well as the overall percentage of availability of medicines and medical supplies. Additionally, the district pharmacist reported that the current availability of medicines and medical supplies was 92%. The reported percentage was according to District Health Information System 2 (DHIS2).

**Table 1:** The average of availability of medicines and medical supplies to the selected public health facilities

Health facilities	Reported %
HF1	90
HF2	90
HF3	70
HF4	90
HF5	90
HF6	90
HF7	70
HF8	50
HF9	70
HF10	70
HF11	70
HF12	90
HF13	90
HF14	70
HF15	70
HF16	70
HF17	70
HF18	70
HF19	90
HF20	70
HF21	70
HF22	50
HF23	70
HF24	70
HF25	70
Total	1870
Average	1870/25 = 74.8

*Source*: Field Data (2022)

On the increased availability of medicines and medical supplies after outsourcing medicines and medical supplies from the PVS, the findings were obtained from 25 health facility in-charges. About 10 (40%) of the health facility in-charges reported that the availability of medicines and medical supplies had been increased at the selected public health facilities, whereas 15 (60%) of the health facility in charges reported that there had been no increase in the availability of medicines and medical supplies at the selected public health facilities. The results are indicated in Table 2.

**Table 2:** Response on increased availability of medicines and medical supplies

		Frequency	Percentage	Valid percentage	Cumulative percentage
Increased availability of medicines and medical supplies	Yes	10	40.0	40.0	40.0
	No	15	60.0	60.0	100.0
	Total	25	100.0	100.0	

*Source*: Field Data (2022)

The assessment on the availability of current stock of medicines and medical supplies at the selected public health facilities revealed that 19 (76%) health facilities were reported having some of the essential medicines and medical supplies from their current stocks, while 6 (24%) assessed health facilities were reported having no current stock of any essential medicines and medical supplies. Table 3 shows the response from the health facility in charges regarding the availability of the current stock of medicines and medical supplies.

**Table 3:** Availability of enough current stock of medicines and medical supplies in health facilities

		Frequency	Percentage	Valid percentage	Cumulative percentage
Availability of enough current stock	Yes	19	76.0	76.0	76.0
	No	6	24.0	24.0	100.0
	Total	25	100.0	100.0	

*Source*: Field Data (2022)

The findings on the challenges of shortage of medicines and medical supplies showed that 20 (80%) health facilities have experience of facing the challenge of shortage of medicines and medical supplies, while only 5 (20%) health facilities were reported that they have not experienced any challenge of shortage of medicines and supplies. Table 4 shows the response on health facilities regarding the challenges of shortage of medicines and medical supplies.

**Table 4:** Status on access to medicines and medical supplies

		Frequency	Percentage	Valid percentage	Cumulative percentage
Challenges of shortage of medicines and medical supplies	Yes	20	80.0	80.0	80.0
	No	5	20.0	20.0	100.0
	Total	25	100.0	100.0	

*Source*: Field Data (2022)

On assessment of patients’ access to essential medicines, the findings showed that 31 (62%) respondents reported to have no full access to essential medicines. The total of 19 (38%) interviewed patients reported having full access to medicines. Furthermore, 46 (92%) interviewed patients had been told to go and buy some essential medicines outside of the health facilities. About 4(8%) interviewed patients from the study sample had not been told about the missing of some essential medicines and were told to go and buy them outside of the health facilities they attended. Tables 5 and 6 show the response of patients regarding access to essential medicines from public health facilities.

**Table 5:** Access to medicines by patients at the public health facilities

		Frequency	Percentage	Valid percentage	Cumulative percentage
Access to essential medicines	Yes	19	38.0	38.0	38.0
	No	31	62.0	62.0	100.0
	Total	50	100.0	100.0	

*Source*: Field Data (2022)

**Table 6:** Patients experience on buying medicines out of the public health facilities

		Frequency	Percentage	Valid percentage	Cumulative percentage
Buying medicines out of the public health facilities	Yes	46	92.0	92.0	92.0
	No	4	8.0	8.0	100.0
	Total	50	100.0	100.0	

*Source*: Field Data (2022)

### The effectiveness of the PVS

This section sought to assess whether PVS has been effective in increasing the availability of medicines and medical supplies to public health facilities. It was assessed by focusing on conformation to the placed orders from the health facilities; timely preparation of orders of medicines and medical supplies; satisfaction on the operation of PVS; adherence to the agreed terms and supply competition.

Regarding conformation to orders from the health facilities, the findings obtained from 27 respondents, including health facility in-charges, district pharmacist, and the contracted prime vendor, showed that 17 (63%) respondents reported inaccuracies in the orders placed from the prime vendor (non-conformation to the orders placed), while 10 (37%) respondents reported accuracy of the placed orders from the prime vendor (conformation to the placed orders). Table 7 shows the response on conformation to the placed orders.

**Table 7:** PVS conforming to the place orders

		Frequency	Percentage	Valid percentage	Cumulative percentage
PVS conforming to the place orders	Yes	10	37.0	37.0	37.0
	No	17	63.0	63.0	100.0
	Total	27	100.0	100.0	

*Source*: Field Data (2022)

In terms of timely preparation of orders of medicines and medical supplies, about 15 (56%) respondents reported a delay in obtaining orders from the prime vendor, whereas 12 (44%) respondents reported no delay in obtaining orders from the prime vendor. Table 8 shows the response on timely preparation of orders.

**Table 8:** Timely preparation of orders

		Frequency	Percentage	Valid percentage	Cumulative percentage
Timely preparation of orders	Yes	12	44.0	44.0	44.0
	No	15	56.0	56.0	100.0
	Total	27	100.0	100.0	

*Source*: Field Data (2022)

The findings under the satisfaction of the operation of PVS were obtained from 26 respondents, including the health facility in-charges and the district pharmacist. The findings revealed as follows: only 1 (4%) respondent reported being not satisfied with the operation of PVS. 20 (77%) respondents reported being slightly satisfied with the operation of PVS, whereby 5 (19%) respondents reported being very satisfied with the operation of PVS. Table 9 displays the responses on the satisfaction on the operation of the PVS.

**Table 9:** Satisfaction on the operation of the PVS

		Frequency	Percentage	Valid percentage	Cumulative percentage
Satisfaction on the operation of the PVS	Not satisfied	1	4.0	4.0	4.0
	Slightly satisfied	20	77.0	77.0	81.0
	Very satisfied	5	19.0	29.0	100.0
	Total	26	100.0	100.0	

*Source*: Field Data (2022)

Adherence to the terms and conditions: only 3(11%) respondents reported that the PVS is able to adhere to the agreed terms, whereas 24 (89%) respondents reported that the PVS is not able to adhere to the agreed terms. Description is presented in Table 10.

**Table 10:** Adherence to the agreed terms

		Frequency	Percentage	Valid percentage	Cumulative percentage
Adherence to the agreed terms	Yes	3	11.0	11.0	11.0
	No	24	89.0	89.0	100.0
	Total	27	100.0	100.0	

*Source*: Field Data (2022)

About the supply competition, the finding gathered from 27 respondents which included 25 health facility in-charges plus the district pharmacist and the prime vendor showed that 1 (4%) respondent reported that there was supply competition against the contracted prime vendor, while 26 (96%) respondents reported that there was no supply competition against the contracted prime vendor. Table 11 shows the response on the operation of the PVS.

**Table 11:** Competition in supply of medicines and medical supplies

		Frequency	Percentage	Valid percentage	Cumulative percentage
Supply competition	Yes	1	4.0	4.0	4.0
	No	26	96.0	96.0	100.0
	Total	27	100.0	100.0	

*Source*: Field Data (2022)

### Qualitative findings

Following the implementation of PVS and its effectiveness on the availability of medicine and medical supplies in public health facilities, respondents thought that there were still inadequate supplies. This was testified by the health facility in charge.

……I cannot say that there is an increase of medicines and medical supplies because I have not seen it to my facility. At this facility the situation is still the same. Even when I receive medicines and medical supplies from MSD I still receive in deficiency compared to the demand of this health facility. Therefore, I cannot say that there is an increase of medicines and medical supplies while I don’t have adequate stock to satisfy the patients.

Another patient added that:

……as a public health facility, I am supposed to access all medical services from this health facility. I remember last time I brought my pregnant wife here to do laboratory tests because they said that it is free, but I was told that some laboratory tests are not available here, so I was asked to take my wife to other health facility where my wife can be tested.

Regarding the operation of the PVS, a health facility in-charge remarked:

……The most serious issue with the contracted prime vendor is lack of some of medicines and medical supplies. Sometimes the health facilities have money, but when you go to the prime vendor you find that no medicines and medical supplies intended to be purchased. At this situation, the prime vendor does not bother to find the alternative ways of obtaining the missing items. Therefore, I do not see the reason of still contracting the existing vendor who is not able to help us. The council should contract the new one who can meet the demand of the health facilities.

A Prime Vendor had the following to say:

……Some of the health facilities are not paying their bills on time.

## Discussion

This study aimed to assess the effectiveness of the PVS on the availability of medicines and medical supplies in selected public health facilities. We found that, PVS is not completely effective in supplying medicines and medical supplies due to its low capacity to conform to the orders placed by the public health facilities, a lack of supply competition, and a failure to adhere to contractual terms.

Regarding the status of the availability of medicines and medical supplies, we found that 74.8% marked the current averages of the availability of medicines and medical supplies, and 60% of the respondents reported that there is no increase on availability of medicines and medical supplies. It has also been reported that 80% of the public health facilities are still facing shortages of medicines and medical supplies, while 76% of the assessed health facilities had stocks of some of the essential medicines and medical supplies, and 24% of the facilities had no stock of medicines and medical supplies. About 62% of patients reported having no full access to essential medicines, whereas 92% of the patients have been told to go and buy some essential medicines outside of the public health facilities.

Of the operation of PVS, 63% of the respondents reported that the PVS does not conform to the placed orders, whereas 56% of the respondents reported the delayed time of preparation of placed orders. About 77% of the respondents were slightly satisfied with the operation of the PVS, whereas 89% of the respondents reported that the PVS was not able to adhere to the agreed terms. Moreover, 96% of the respondents, which included health facility in-charges and district pharmacist, reported that there was no supply competition against the contracted prime vendor.

The findings of this study have shown that there is a shortage of medicines and medical supplies to the selected public health facilities as reported by 80% of respondents. However, the reported findings is in contradiction with the study conducted by [15] which reported an increase in the availability of medicines and medical supplies and this was attributed to the operation of PVS. On the other hand, the percentage of availability of medicines and medical supplies (74.8%) reported by this study, is not very far from the average percentage (78.4%) reported by [20] a recent unpublished study. The studies by [9, 21, 22] evidenced that there are still challenges of shortage of medicines and medical supplies in the health systems. The decreased availability of medicines and medical supplies affected the access to essential medicines and current stocks.

Regarding the capacity of prime vendor to fulfil medicine and medical supply orders, the study found the capacity of PVS to be low due to a failure in conformation to the orders placed as well as delays in preparing the orders placed resulting in dissatisfied operations. The low capacity has been influenced by increase in demand that has overwhelmed the supply capacity of the contracted prime vendor as there is only one prime vendor per region regardless of number of public health facilities. The delay in preparation of orders caused the public health facilities to operate with missing items, hence; shortage of medicines and medical supplies. Under this situation, most of patients lack access to essential medicines whereas healthcare workers lack medical supplies such as gloves, syringes and some of the laboratory test kits.

Lack of supply competition among prime vendors decreases the effectiveness of the PVS which leads to dissatisfaction of the customers [23]. Supply competition provides an opportunity for the customers to make judgment on the products such as price comparison as well as quality assessment of the products [23, 24]. Supply competition is very important as it controls the price inflation and maintains discipline of suppliers to value customers. Lack of supply competition may result in increased prices of the health commodities [25].

The findings noticed a low capacity on adhering to the contracted terms. The contracted prime vendor neither provides prior notices on a change of prices nor directs the delivery of medicines and medical supplies to the public health facilities as per agreement. Failure to provide notification regarding the change of prices disturbs the budget of the public health facilities. However, most of the public health facilities were reported of not paying for medicines and medical supplies on time. This poses a challenge to the operation of PVS. According to respondents, public health facilities procure medicines and medical supplies by credit, but they fail to maintain payment schedule as per the agreement.

Regarding the direct delivery of health commodities to the public health facilities, PVS does not make direct deliveries of medicines and medical supplies to the public health facilities. The failure of direct delivery of health commodities to the public health facilities is due to the fact that the public health facilities do not pay transportation fees.

### Limitation of the study and future research

The study was limited by inability to access some of the important information regarding the operation of prime vendors. Also, some of the respondents were not ready to disclose some information. Further studies should be done to evaluate the accountability of the public health facilities in the partnership with the PVS.

## Conclusion

Despite the fact that the operation of PVS created several challenges in terms of medicine and medical supply availability, the system has aided in the availability of medicines and medical supplies to the selected public health facilities. The challenges discovered in this study are correctable, allowing each side to benefit from the operation of the PVS. The discovered challenges include low capacity for conforming to the placed orders of medicines and medical supplies from public health facilities; lack of supply competition; and poor adherence to the contracted terms. However, the contracted prime vendor reported that some of the health facilities have negative support on the operation of PVS since the facilities delay on paying bills, which then leads to the accumulation of the debits. Therefore, there is a need for a constructive relationship between PVS and public health facilities to reinforce adherence to the contracted terms.

## Data Availability

All the data used for this study are available upon a reasonable request from the first author.

## Abbreviations

DHIS2: District Health Information System 2
MoH: Ministry of Health
MSD: Medical Stores Department
PPP: Public-Private partnership
PVS: Prime Vendor System
SPSS: Statistical Package for Social Sciences
O/S: Out of Stock
